# Inhibition of Fibroblast Growth by Notch1 Signaling Is Mediated by Induction of Wnt11-Dependent WISP-1

**DOI:** 10.1371/journal.pone.0038811

**Published:** 2012-06-08

**Authors:** Zhao-Jun Liu, Yan Li, Yurong Tan, Min Xiao, Jialin Zhang, Freddy Radtke, Omaida C. Velazquez

**Affiliations:** 1 Department of Surgery, Miller School of Medicine, University of Miami, Miami, Florida, United States of America; 2 Sylvester Comprehensive Cancer Center, University of Miami, Miami, Florida, United States of America; 3 The Wistar Institute, Philadelphia, Pennsylvania, United States of America; 4 Ecole Polytechnique Fédérale de Lausanne, Swiss Institute for Experimental Cancer Research, Switzerland; University of Maastricht (UM), The Netherlands

## Abstract

Fibroblasts are an integral component of stroma and important source of growth factors and extracellular matrix (ECM). They play a prominent role in maintaining tissue homeostasis and in wound healing and tumor growth. Notch signaling regulates biological function in a variety of cells. To elucidate the physiological function of Notch signaling in fibroblasts, we ablated *Notch1* in mouse (*Notch1^Flox/Flox^*) embryonic fibroblasts (MEFs). Notch1-deficient (*Notch1* ^−/−^) MEFs displayed faster growth and motility rate compared to *Notch1^Flox/Flox^* MEFs. Such phenotypic changes, however, were reversible by reconstitution of Notch1 activation via overexpression of the intracellular domain of Notch1 (NICD1) in Notch1-deficient MEFs. In contrast, constitutive activation of Notch1 signaling by introducing NICD1 into primary human dermal fibroblasts (FF2441), which caused pan-Notch activation, inhibited cell growth and motility, whereas cellular inhibition was relievable when the Notch activation was countered with dominant-negative mutant of Master-mind like 1 (DN-MAML-1). Functionally, “Notch-activated” stromal fibroblasts could inhibit tumor cell growth/invasion. Moreover, Notch activation induced expression of Wnt-induced secreted proteins-1 (WISP-1/CCN4) in FF2441 cells while deletion of *Notch1* in MEFs resulted in an opposite effect. Notably, WISP-1 suppressed fibroblast proliferation, and was responsible for mediating Notch1's inhibitory effect since siRNA-mediated blockade of WISP-1 expression could relieve cell growth inhibition. Notch1-induced WISP-1 expression appeared to be Wnt11-dependent, but Wnt1-independent. Blockade of Wnt11 expression resulted in decreased WISP-1 expression and liberated Notch-induced cell growth inhibition. These findings indicated that inhibition of fibroblast proliferation by Notch pathway activation is mediated, at least in part, through regulating Wnt1-independent, but Wnt11-dependent WISP-1 expression.

## Introduction

Fibroblasts are key components of the interstitial tissue present in most organs of the body [1]. They provide a delicately balanced tissue-specific ECM that partitions the interstitial space between tissue cells, blood vessels and nerves. Fibroblasts play an important role in not only supporting tissue architecture, but also participating in maintenance of tissue homeostasis. Fibroblasts generate soluble proteins including growth and differentiation factors [2] and remodelling enzymes, for example, matrix metalloproteases (MMPs) [3]. These important cells are also involved in synthesis of ECM, such as collagen and fibronectin [4]. Fibroblasts are known to play a role in a variety of fibrotic disorders (fibrosis/sclerosis). Most recently, these cells have gained increasing attention since they are important components of the supporting stroma in a variety of solid tumors. Tumors have been characterized as a type of “wound that does not heal” [5] and are now viewed as “organs” which have a unique microenvironment and specific stromal compartment. Tumor stroma is comprised of inflammatory cells, endothelial cells, fibroblasts and ECM. Fibroblasts in tumor tissues have been termed carcinoma-associated fibroblasts (CAFs), tumor-associated fibroblasts (TAFs) or cancer-associated fibroblasts (CAFs) (herein termed as cancer-associated fibroblasts (CAFs)) [6]. CAFs are postulated to promote tumor growth through direct stimulation of tumor cell proliferation and promotion of tumor angiogenesis. Fibroblasts, thus, may represent a new therapeutic target for modulating stroma-associated tissue regeneration and tumor growth.

In normal adult tissue, resident fibroblasts are maintained in a relatively quiescent state in which they are involved in slow turnover of the ECM. Fibroblasts, once activated, undergo a change in phenotype from the quiescent state to a proliferative and contractile phenotype termed myofibroblasts (sometimes termed “activated fibroblasts”). Myofibroblasts actively produce growth factors and ECM, display an elongated spindle shape, and express contractile α–smooth muscle actin (α-SMA) and vimentin [7]. Myofibroblasts can arise from the local, resident fibroblasts or from circulating mesenchymal precursors/stem cells [8], and even from epithelial cells via epithelial mesenchymal transition (EMT) [9].

The Notch signaling pathway is an evolutionarily conserved signaling cascade that regulates a variety of cellular activities including proliferation, differentiation, quiescence and death [10]. The Notch receptor and its ligands are transmembrane proteins whose signaling requires cell to cell contact between neighboring cells. Mammals have four Notch receptors (Notch1–4) and five Notch ligands which fall into two classes: Delta-like (Dll) and Jagged. Activation of Notch receptors is triggered by interaction with Notch ligands on adjacent cells. The receptor-ligand binding results in proteolytic cleavage (by TACE and γ-secretase) of NICD from the membrane bond Notch. NICD subsequently translocates into the nucleus where it binds to CSL (CBF1/Suppressor of Hairless/Lag-1)/RBP-Jκ and recruits Mastermind-like (MAML) to form a ternary complex that functions as a transcriptional activator of Notch target genes. Notch target genes include those belonging to the *Hes* and *Hey* families [11]. The diverse outcome of Notch activation is dependent on several factors including the specific timing, the signal strength/gene dosage, and the cell type and context [12]–[14].

The role of Notch signaling in fibroblasts is poorly studied. In this work we investigated the function of Notch signaling in regulating the cell growth of fibroblasts through *in vitro* loss-/gain-of-function approaches. We observed a suppressive effect of activation of Notch signaling on fibroblast proliferation. We demonstrated that the inhibitory effect of Notch signaling is partially mediated by the induction of WISP-1 (CCN4) through a Wnt11-dependent mechanism in fibroblasts.

## Results

### Deletion of Notch1 Increases Cell Growth and Motility of MEFs

**Figure 1:**
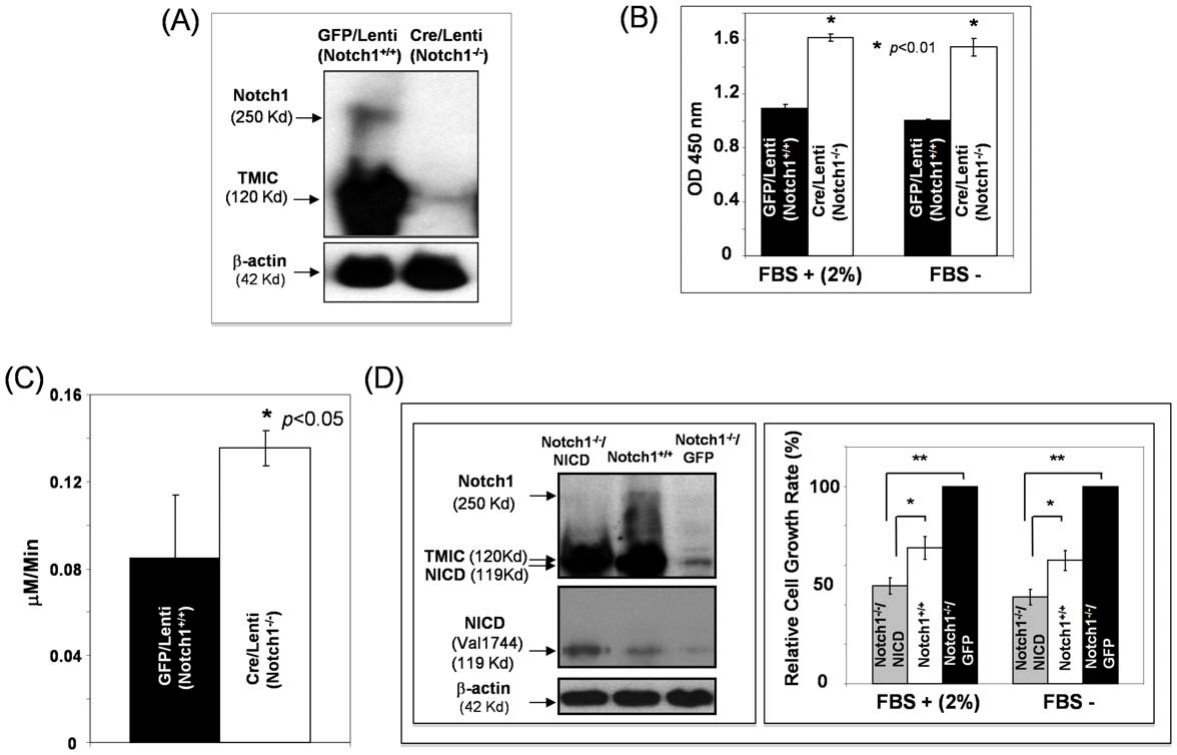
Deletion of Notch1 relieves its suppressive effect on cell growth and motility of MEFs. (**A**) Expression of full length Notch1 (250 Kd) and Trans-Membrane and Intra-Cellular domain (TMIC, 120 Kd) in Cre/lenti- versus GFP/Lenti-transduced *Notch1^Flox/Flox^* MEFs. Knock-out Notch1 in Cre/lenti-transduced *Notch1^Flox/Flox^* MEFs was analyzed by Western blotting assay. β-actin was used as loading control. (**B**) Rate of cell growth of MEFs in the presence 2% or absence of serum was measured by MTT assay. 5,000 cells/well were plated. Deletion of Notch1 promotes cell growth of MEFs. (**C**) Deletion of Notch1 increases cell motility of MEFs. The migration of 15 randomly selected cell pairs was tracked over time (20 hours) by time-lapse photography and velocity was calculated with software. (**D**) *Left*: Expression of endogenous full length Notch1 (250 Kd), TMIC (120 Kd) and exogenous NICD (119 Kd) in MEFs was shown. Expression of activated Notch1 (NICD) was detected by specific antibody recognizing Val1744. *Right*: Increased cell growth rate was reversed by reconstituted Notch1 activation in Notch1-deficint (*Notch1 ^−/−^*) MEFs by NICD overexpression as demonstrated by immunoblotting (β-actin was used as loading control). Cell growth rate of MEFs in the presence 2% or absence of serum was measured by MTT assay. * *P*<0.05; ** *P*<0.01. Data are presented as mean ± SD of three independently performed experiments in (B), (C), and (D).

To study the physiological function of Notch1 in regulating fibroblast proliferation and migration, we deleted *Notch1* gene in MEFs isolated from *Notch1^Flox/Flox^* mouse embryos at E13.5 by transducing cells with Cre/lentiviruses versus GFP/lentiviruses (control) *in vitro*. Greater than 95% cells were transduced based on the percentage of GFP-positive cells observed under fluorescence microscopy. A similar percentage of Cre-positive cells were achieved using same MOI of Cre/lentiviruses (data not shown). Knock-down of Notch1 was confirmed by Western blot analysis (Figure 1A) a week after transduction. A small residual Notch1 expression observed can be explained by non-100% transduction of MEFs by Cre/lentiviruses. Notch1-deficient (*Notch1* ^−/−^) MEFs displayed faster cell growth rate as measured by MTT assay compared to (*Notch1^Flox/Flox^*) MEFs (Figure 1B). Cell morphology of the *Notch1* ^−/−^ MEFs appeared to be unaltered compared to the *Notch1 ^Flox/Flox^* MEFs. Cell motility was increased in Notch1-deficient (*Notch1* ^−/−^) versus *Notch1 ^Flox/Flox^* MEFs as demonstrated by recording the trafficking of 15 randomly selected MEFs for 20 hours in an *in vitro* cell scratch assay (Figure 1C). To functionally validate the phenotypes observed in Notch1-deficient (*Notch1* ^−/−^) MEFs, we transduced these cells with NICD (an active form of Notch1) and GFP (control), respectively, using lentiviral vectors to reconstitute Notch1 activation. It was observed that phenotypic changes resulted from Notch1 deletion were reversible by reconstitution of Notch1 activation (Figure 1D). Notably, constitutive activation of the Notch1 pathway by NICD overexpression even resulted in a significant growth inhibition of Notch1 null MEFs (*Notch1^−/−^*) compared to wild type of MEFs (*Notch1^+/+^*). This is consistent with what we observed in human dermal fibroblasts (FF2441) (see below). Overall, these results indicate that Notch1 signaling in MEFs exerts a suppressive effect on cell growth and motility.

### Constitutive Expression of Exogenous NICD1 Induces Pan-Notch Activation in Human Fibroblasts

**Figure 2:**
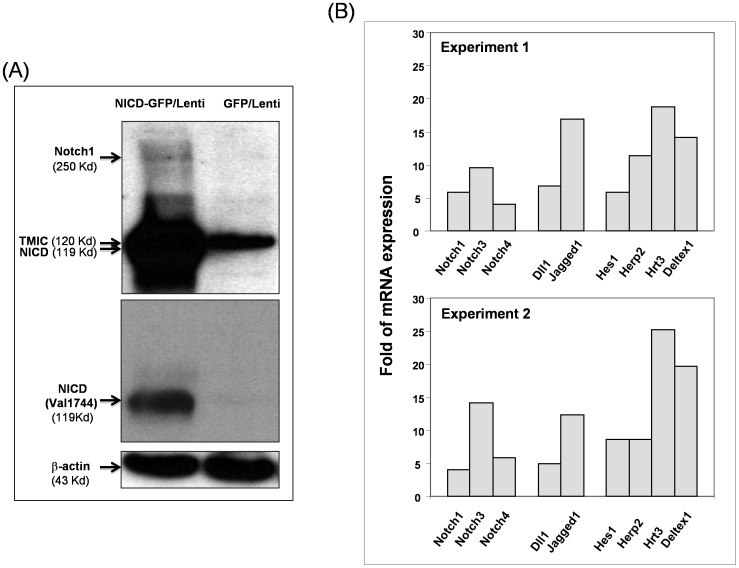
Activated Notch1 induces pan-Notch activation in human fibroblasts. (**A**) FF2441 cells were transduced with either NICD1-GFP/lenti or GFP/lenti. Two days after transduction, transductants were harvested and subjected to Western blot analysis. Expression of endogenous full length Notch1 (250 Kd), TMIC (120 Kd) and exogenous NICD (119 Kd) in MEFs was shown. Expression of activated Notch1 (NICD) was detected by specific antibody recognizing Val1744. β-actin was used as control. (**B**) Constitutive activation of Notch1 results in self-propagated pan-Notch activation. Up-regulation of Notch receptors, ligands and target genes in FF2441-NICD1-GFP versus FF2441-GFP is observed by RT^2^ PCR Array. The folds of increased gene expression in FF2441-NICD1-GFP compared to FF2441-GFP cells from two independently performed experiments were shown.

To further study the role of Notch signaling activation on cell growth of fibroblasts, we aimed to examine the effects of constitutive Notch1 activation on proliferation of primary human dermal fibroblasts (FF2441) [14]. To this end, we transduced FF2441 cells with lentiviral vectors encoding either the *GFP* marker gene or the *NICD1* gene linked to the *GFP* marker gene via internal ribosome entry site (*IRES*) to express GFP and NICD1 independently [15]. The NICD1–GFP-transduced fibroblasts simultaneously expressed NICD1 and GFP. At an MOI of 5, greater than 95% of transduced fibroblasts expressed GFP as observed by fluorescence microscopy (data not shown). Expression of NICD1 in transduced FF2441 cells was confirmed by immunoblotting analysis (Figure 2A). Interestingly, full-length of Notch1 (∼250 Kd) was undetectable in fibroblasts transduced with GFP/lenti, while detectable in fibroblasts transduced with NICD-GFP/lenti. It suggested that constitutive expression of exogenous NICD induced expression of endogenous Notch1. We further examined the gene profiles of Notch pathway components using PCR array, we observed a “self-propagated” pan-Notch activation mechanism induced by enforced expression of exogenous NICD1 in human fibroblasts. We found that constitutive expression of NICD1 resulted in the up-regulation of gene expression of multiple Notch pathway components in FF2441 cells, including ligands (*Dll1*, *Jagged1*), receptors (*Notch1, 3*, *and 4*), and target genes (*Deltex1*, *Hes1*, *Herp2*, and *Hrt3*). The changes in gene induction were showed in Figure 2B. Thus, hyper-activation of Notch1 pathway caused a pan-Notch signaling activation. These results thus revealed a “self-propagated” pan-Notch activation mechanism induced by enforced expression of exogenous NICD1 in human fibroblasts.

### Enforced Activation of Notch Pathway Inhibits Human Fibroblast Proliferation, but Did Not Induce Cell Apoptosis

**Figure 3:**
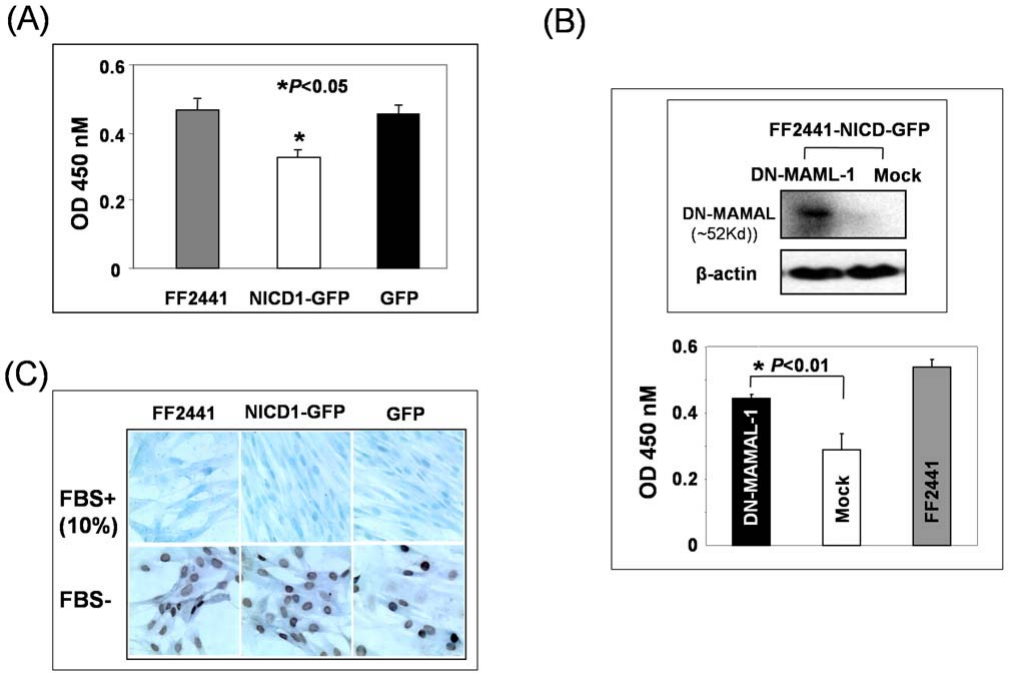
Effects of Notch activation and inhibition on human dermal fibroblast growth. Notch activation slows down cell growth rate of fibroblasts. Proliferation of transduced FF2441 cells was determined by MTT assays (**A**). Activated Notch1 inhibited cell growth of fibroblasts. (**B**) Inhibition of Notch activation by ectopic expression of DN-MAML-1 (*upper*) in FF2441-NICD-GFP cells relieves cell growth inhibition as measured by MTT assay (*lower*). Data are presented as mean ± SD of three independently performed experiments in (A) and (B). (**C**) Notch activation does not induce cell apoptosis in fibroblasts. Cell apoptosis was determined by TUNEL assay. No obvious apoptotic cells were detectable in FF2441-NICD1-GFP cells. Serum starvation-induced cells were used as positive control (FBS-).

We therefore investigated the effect of enforced activation of Notch1 pathway on cell biology of human fibroblasts. The constitutive activation of Notch1 by introduction of NICD into FF2441 cells significantly inhibited cell growth and motility in human dermal fibroblasts. As determined by MTT assay, cell proliferation rate of FF2441-NICD1–GFP significantly decreased compared to FF2441-GFP cells (Figure 3A). However, such a phenotypic change was reversible when the Notch activation was countered with DN-MAML-1, an antagonist of Notch pathway activation. As shown in Figure 3B, cell growth rate of FF2441-NICD1–GFP-DN-MAML-1 was significantly re-boosted compared to the control (FF2441-NICD1–GFP- Mock). Moreover, Notch pathway activation retarded the cell growth of FF2441, but did not induce cell apoptosis as no increase in apoptotic cells was detectable by TUNEL assay (Figure 3C). Constitutive Notch pathway activation down-regulated expression a panel of cell cycle genes, including *cyclin D2*, *cyclin C*, *cycling E1* and *cdk 4*, *6*, *7* and *8* while up-regulated expression of *CDK inhibitor 3* (Table 1), suggesting that Notch pathway activation results in cell cycle arrest in human dermal fibroblasts. Similarly, as determined by time-lapse photography to track and measure cell trafficking in a modified scratch assay (*in vitro* wound healing assay), the average distance traveled by cells toward the middle line of the gap was smaller at all time points for FF2441-NICD1–GFP compared to FF2441-GFP cells. Velocity of FF2441-NICD1–GFP versus FF2441-GFP cells was 14.8+/−4.9 (mean +/− SD) versus 35.9+/−6.1 µm/hour (data not shown). These observations indicated that constitutive activation of Notch pathway inhibits cell proliferation and migration in human dermal fibroblasts. Taken together, the above experiments revealed that deletion of Notch1 increases cell growth and motility in MEFs, while activation of Notch pathway inhibits cell growth and motility in human dermal fibroblasts. These data consistently pointed to a role of Notch signaling in modulating the growth and motility of fibroblast cells.

**Table 1:** Differential expression of genes involved in cell cycle control between NICD1-GFP/FF2441 and GFP/FF2441 cells.

Genes	Fold Changes of Gene Expression in NICD1-GFP/FF2441 *vs* GFP/FF2441 Cells (Mean ± SD)
*Cyclin D2*	−2.88±0.08
*Cyclin C*	−3.36±0.12
*Cyclin E1*	−4.74±0.10
*CDK 4*	−2.18±0.08
*CDK 6*	−6.00±0.15
*CDK 7*	−3.86±0.06
*CDK 8*	−2.24±0.08
*CDK Inhibitor 3*	+2.28±0.07

### Status of Notch Signaling Is Correlated with the Biological Activity of Fibroblasts

**Figure 4:**
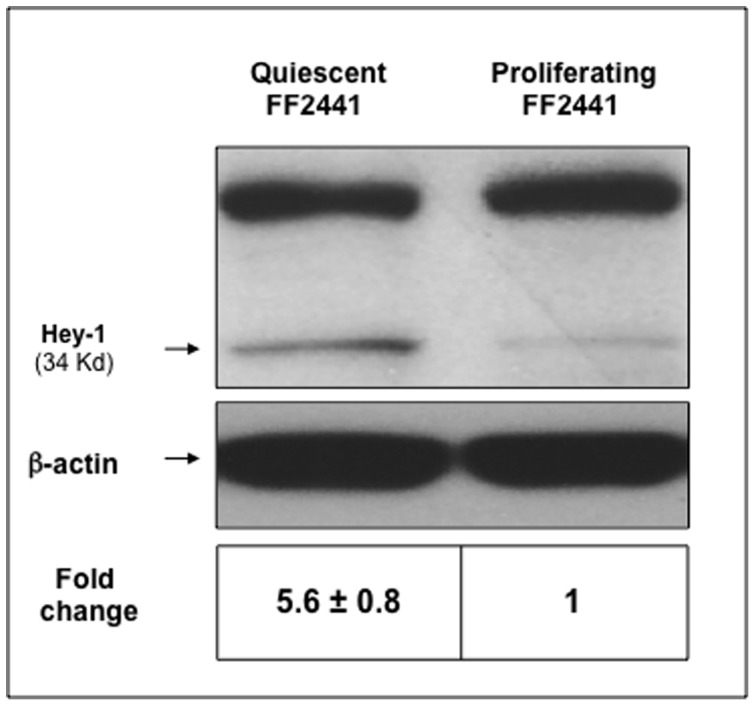
The Notch pathway is maintained in a less-activated or inactivated status in proliferating human dermal fibroblasts. The increased expression of Hey-1 protein in quiescent fibroblasts compared to proliferating fibroblasts was demonstrated by immunoblotting analysis. β-actin was used as loading control.

To further study the correlation between the Notch pathway activation and the biological activity of fibroblasts, we examined the status of Notch signaling in cultured, proliferating FF2441, which were basically “activated” due to stimulation of serum and growth factors in the culture medium, versus quiescent FF2441 cells, which were induced by cell-contact inhibition and serum starvation. The expression of Notch receptors, ligands and known target genes (including Hes and Hey) were analyzed by the RT° Profiler™ PCR Array system. None of the Notch signaling pathway genes was significantly expressed in the proliferating fibroblasts. In drastic contrast, the expression of *Dll-1*, *Jagged-2*, *Notch1*, *Notch-3*, *Notch-4*, *TACE* (*ADAM-17*) and *Hey-1* were significantly up-regulated (>1.5-fold) in quiescent fibroblasts (Table 2). The increased protein expression of Hey-1 (in quiescent compared to proliferating fibroblasts) was confirmed by immunoblotting analysis (Figure 4). These data indicate that the Notch pathway is maintained in a less-activated or inactivated status in proliferating human dermal fibroblasts whereas when these cells become quiescent, they upregulate several Notch signaling components. Thus, the status of Notch signaling appears to be tightly correlated with the biological activity of fibroblasts.

**Table 2:** Notch pathway is activated in quiescent fibroblasts.

Genes	Fold Increase of Gene Expression (Quiescent/Proliferating Cells) (Mean ± SD)	*P*-Value
**Notch1**	**1.36±0.05**	**<0.01**
Notch2	0.38±0.04	>0.05
**Notch3**	**1.82±0.21**	**<0.01**
**Notch4**	**2.12±0.46**	**<0.01**
Jagged-1	0.78±0.08	>0.05
**Jagged-2**	**3.46±0.55**	**<0.01**
**Dll-1**	**2.02±0.40**	**<0.01**
Hes-1	0.78±0.06	>0.05
**Hey-1**	**1.56±0.15**	**<0.01**
HeyL	0.24±0.03	>0.05
Presenilin-1	0.56±0.05	>0.05
**TACE**	**2.34±0.32**	**<0.01**

### “Notch-Activated” Stromal Fibroblasts Inhibited Tumor Growth/Invasion in a 3D Skin Melanoma Model

**Figure 5:**
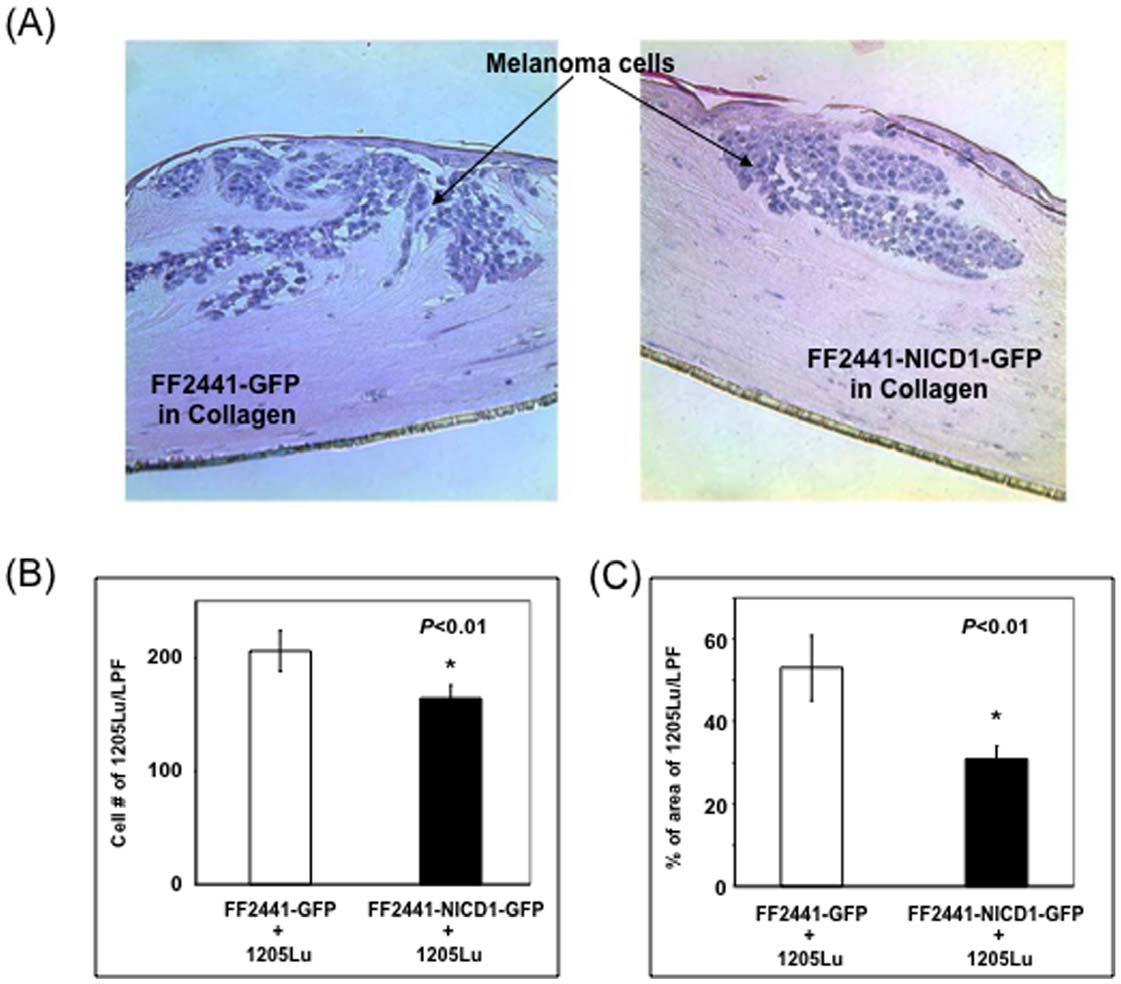
Inhibitory effects of “Notch-activated” fibroblasts as stromal cells on melanoma cell growth and invasion in 3D model. (**A**) Representative images of H&E staining of sections of 3D skin melanoma model. (**B**) Melanoma cell growth in 3D skin model. Significantly decreased melanoma cells were observed in 3D reconstructs in which FF2441-NICD1-GFP cells were embedded in collagen. (**C**) Decreased invasion into ‘dermis’ by melanoma cells in 3D skin reconstructs in which FF2441-NICD1-GFP cells were embedded. All data are calculated based on that from 5 randomly selected LPF/section and totally 10 sections per group.

To address the potential biological relevance of Notch pathway activation in fibroblasts, we tested the role of “Notch-activated” fibroblasts as stromal cells in modulating the growth and invasion/migration of melanoma cells since it is well established that stromal fibroblasts/CAFs play a critical role in regulating tumor growth and metastasis. For this purpose, a 3D skin melanoma reconstruct model, which resembles human physiological condition, was utilized. Skin reconstructs consist of a ‘dermis’ of collagen with embedded fibroblasts (FF2441-NICD1–GFP versus FF2441-GFP) and an ‘epidermis’ of multi-layered keratinocytes with equal number of metastatic melanoma cells (1205Lu) [16] [17]. After 14 days in culture, more 1205Lu cells were countable and many 1205Lu cells were able to invade into ‘dermis’ in which FF2441-GFP were embedded. Strikingly, invasion/migration of melanoma cells into ‘dermis’, in which FF2441-NICD1–GFP were embedded, was remarkably suppressed, and less 1205Lu cells were detectable (Figure 5). These data indicated that “Notch-activated” fibroblasts inhibited growth of melanoma cells in “skin” and their invasion into ‘dermis’.

### Effect of Notch Signaling on Regulating WISP-1/CCN4 Expression in Fibroblasts

**Figure 6:**
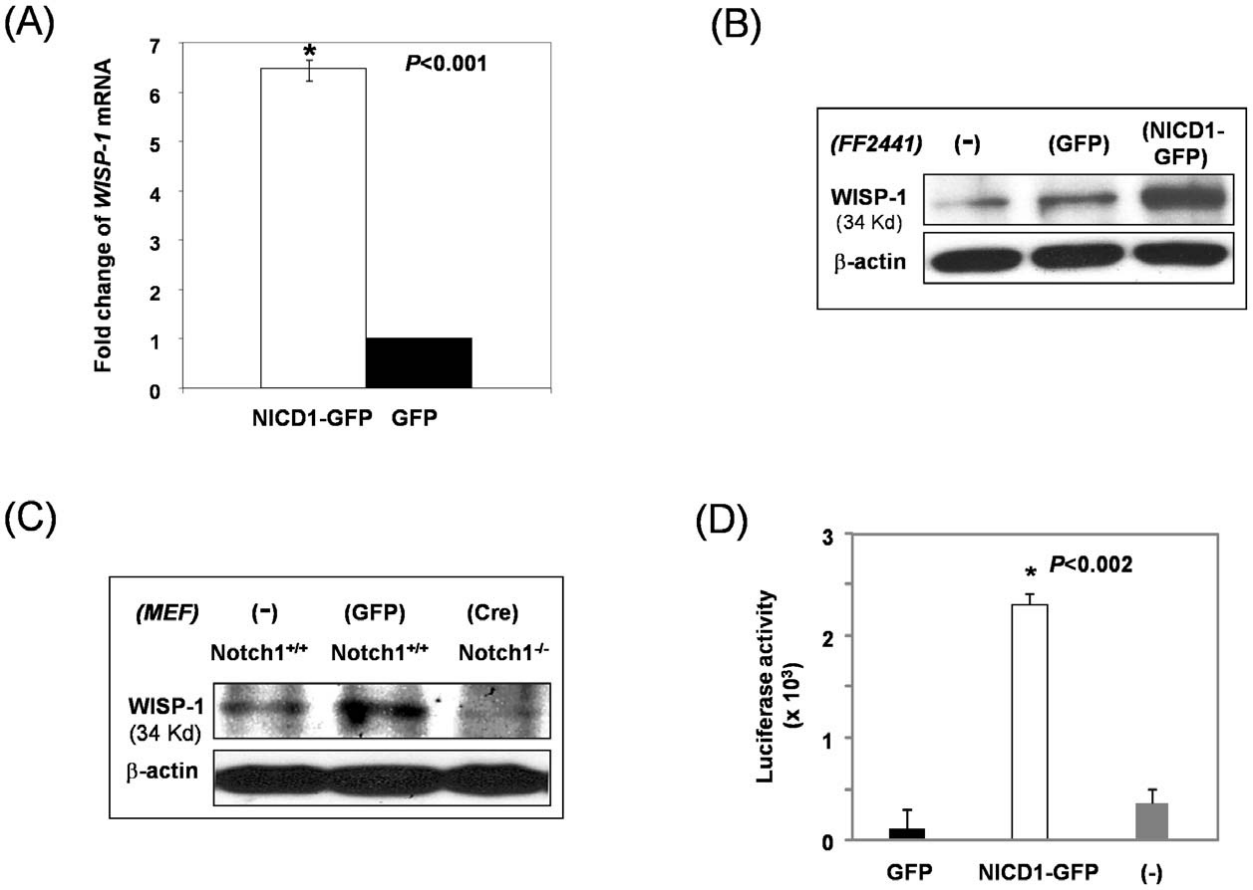
Notch pathway activation induces expression of WISP-1/CCN4 in fibroblasts. (**A**) Levels of mRNA of *WISP-1*/*CCN4* were up-regulated in FF2441-NICD1-GFP compared to FF2441-GFP cells. Data are from RT^2^ PCR Array and presented as fold changed in gene expression by setting levels of genes in FF2441-GFP as “1”. Data are presented as mean ± SD of three independently performed experiments. (**B**) Expression of WISP-1/CCN4 protein was up-regulated by Notch pathway activation in fibroblasts. Expression of WISP-1/CCN4 in FF2441-NICD1-GFP versus FF2441-GFP and untreated F2441 (−) cells was analyzed by Western blotting assay. β-actin was used as loading control. (**C**) Expression of WISP-1/CCN4 protein was down-regulated in *Notch1* ^−/−^ MEFs. Expression of WISP-1/CCN4 in *Notch1* ^−/−^ versus *Notch1^Flox/Flox^* MEFs (both untreated (−) and GFP/lenti-transduced) was analyzed by Western blotting assay. β-actin was used as loading control. (**D**) Increased WISP-1 promotor-driven luciferase activity in FF2441-NICD1-GFP compared to FF2441-GFP cells. Data are presented as mean ± SD of three independently performed experiments.

To understand the signaling mechanisms for the pronounced effects of the Notch signaling on cell biology of fibroblasts, we examined the gene expression profile of Notch pathway activation in FF2441 cells using quantitative Human Notch Signaling Pathway RT^2^ Profiler™ PCR array, and found an approximately 6.5-fold increase in the gene expression of *WISP-1*/*CCN4* in FF2441-NICD1–GFP compared to FF2441-GFP cells (Figure 6A). To validate the observed up-regulation of mRNA of *WISP-1*/*CCN4*, we conducted immunoblotting analyses and confirmed an up-regulated protein expression of WISP-1/CCN4 in FF2441-NICD1–GFP compared to FF2441-GFP cells (Figure 6B). Consistently, levels of WISP-1/CCN4 protein were down-regulated in Notch1-deficient (*Notch1* ^−/−^) MEFs compared to *Notch1^Flox/Flox^* MEFs as demonstrated by immunoblotting analyses (Figure 6C). Transcriptional activation of *WISP-1* gene was also tested in FF2441-NICD1–GFP versus FF2441-GFP cells. Cells were transduced with *WISP1 promoter*-*Luc2*/Lentivirus, control RPL10PROM/Lentivirus and R01_PROM/Lentivirus, respectively, and lucifease activities were measured 48 hours post-transduction. As shown in Figure 6D, constitutive activation of Notch1 significantly increased WISP-1 promoter activity in fibroblasts compared with control. Thus, these experiments identified WISP-1/CCN4 as one of the down-stream targets of Notch pathway in fibroblasts. An *in silico* analysis of CSL binding site in the promoter of WISP-1 gene did not reveal a matching sequence, implicating that Notch indirectly regulates WISP-1 gene expression. This is consistent with our findings showed below that the regulation of WISP-1 by Notch is mediated by Wnt-11 in fibroblasts.

### WISP-1/CCN4 Suppresses Fibroblast Proliferation

**Figure 7:**
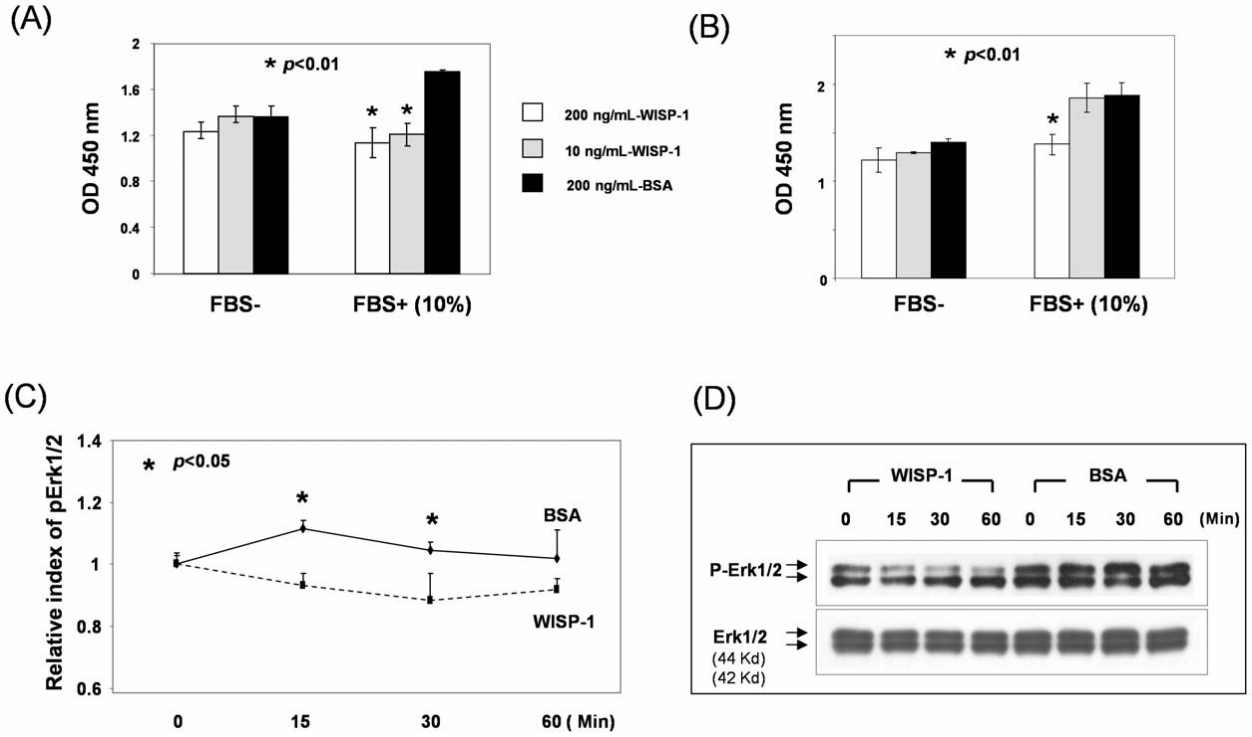
Effect of WISP-1/CCN4 on cell growth of fibroblasts. Supplementation of exogenous recombinant human WISP-1/CCN4 slowed down cell growth of FF2441 cells (**A**) and Cre/MEFs (**B**). Proliferation of cells was determined by MTT assays. 5,000 FF2441 cells/well and 2,000 Cre/MEFs/well were plated respectively. Data are presented as mean ± SD of three independently performed experiments. (**C**) Addition of 200 ng/mL of recombinant human WISP-1/CCN4 inhibited serum-induced phosphorylation of Erk1/2 in FF2441 cells. Total amount of Erk1/2 was used as control. Time course of phosphorylation of Erk1/2. Serum starved cells were stimulated with 10% serum in the presence of WISP-1/CCN4 for varying times. Autophotographs of Western blots were quantified by computerized densitometry. pERK1/2 signals were normalized to total ERK levels and unstimulated (time “0”) samples were set as “1”. Relative index of pErk1/2 compared to unstimulated samples from three independent Western blots was plotted. (**D**) A representative Western blot was shown.

Since constitutive activation of Notch1 pathway resulted in a cell growth inhibition in fibroblasts, we sought to investigate a potential role of WISP-1/CCN4 in mediating Notch-induced growth control of fibroblasts. To examine for a potential biological function of WISP-1/CCN4 in regulating cell growth in fibroblasts, we performed MTT assay to test the effect of recombinant human WISP-1/CCN4 on cell proliferation in FF2441 cells since WISP-1/CCN4 is a soluble factor secreted by cells. Addition of recombinant human WISP-1/CCN4 into the cell culture medium significantly inhibited the growth rate of FF2441 cells, but only in the presence of 10% FBS in the culture medium. In serum-free medium, no significant inhibition was achievable. Two dosages of WISP-1/CCN4 were tested, and both 10 ng/mL and 200 ng/mL achieved comparable inhibition efficacy (Figure 7A). Similarly, supplementation with recombinant human WISP-1/CCN4 significantly slowed cell growth rate of Cre/MEFs in the presence of 10% of FBS, but not in the absence of serum. Only the higher dose (200 ng/mL) of WISP-1/CCN4 was able to suppress cell growth in Cre/MEFs, (Figure 7B), suggesting variable sensitivity of different types of fibroblasts in responding to WISP-1/CCN4. The serum-dependent effect of WISP-1/CCN4 on fibroblast proliferation suggested that WISP-1/CCN4 interferes with growth factor(s) in FBS that induce proliferation. However, WISP-1 does not appear to induce a direct growth inhibition signal. In support of this concept, we determined that addition of recombinant WISP-1/CCN4 (200 ng/mL) inhibited serum-induced phosphorylation of Erk1/2 (the signaling cascade delivered from the MAPK pathway ultimately regulates the cell cycle machinery) in human fibroblasts (Figure 7C, 7D) which were starved overnight with serum-free medium and re-stimulated with FBS (10% in the culture medium) in the presence of WISP-1/CCN4. These results demonstrated a specific serum-dependent inhibitory effect of WISP-1/CCN4 on the cell growth of fibroblasts that has not been previously reported.

### Inhibitory Effect of Notch Signaling on Human Fibroblast Proliferation Is Partially Mediated by WISP-1/CCN4

**Figure 8:**
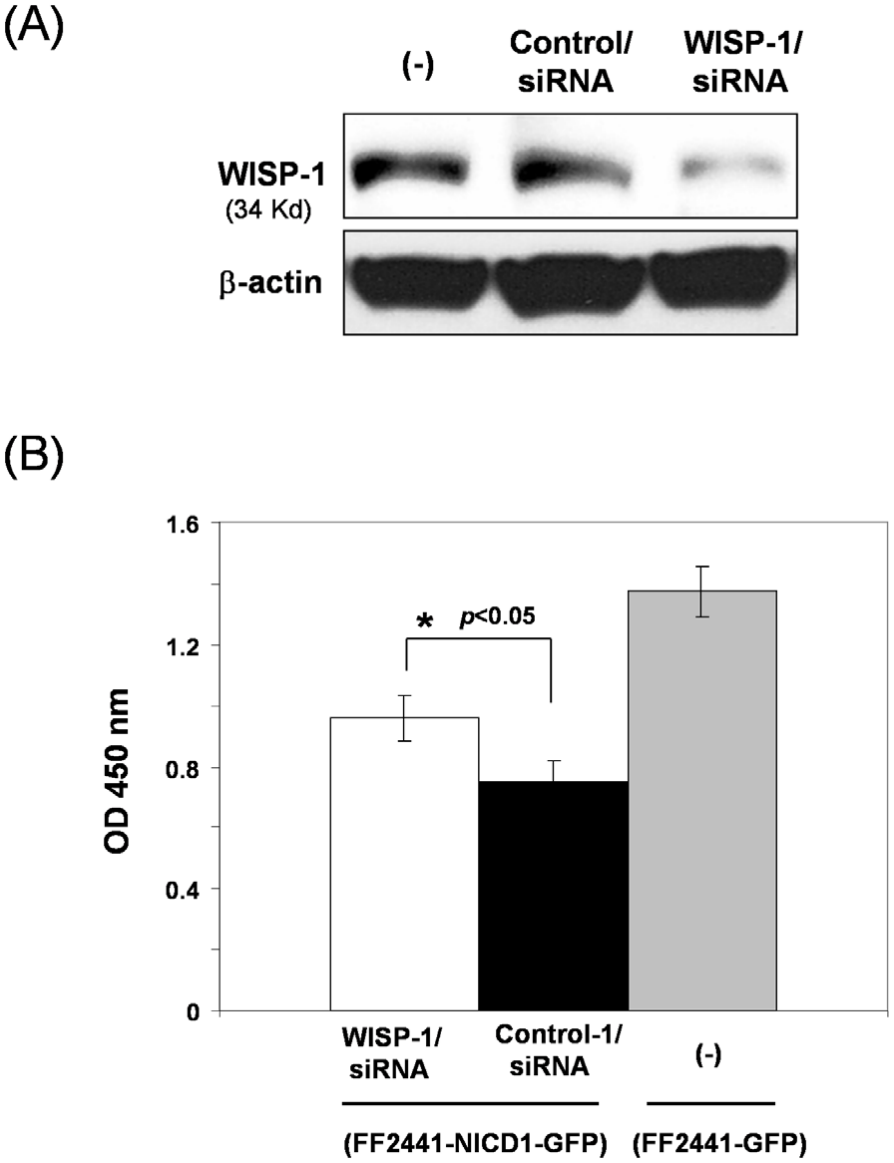
WISP-1/CCN4 is partially responsible for mediating the inhibitory effect of Notch signaling on fibroblast proliferation. (**A**) siRNA-mediated knocking-down WISP-1/CCN4 expression in human fibroblasts. Expression of WISP-1/CCN4 in siRNA-transfected versus non-targeting control siRNA-transfected or untransduced FF2441-NICD1-GFP cells was analyzed by Western blotting assay. β-actin was used as loading control. (**B**) Blockage of WISP-1/CCN4 expression relieved Notch's inhibitory effect on cell growth of fibroblasts. Proliferation of siRNA-transfected versus non-targeting control siRNA-transfected FF2441-NICD1-GFP cells was determined by MTT assays. 2,000 cells/well were plated. Data are presented as mean ± SD of three independently performed experiments.

Based on the findings detailed above, we hypothesized that the inhibitory effect of Notch pathway activation on cell growth of fibroblasts may be mediated through up-regulating the production of WISP-1/CCN4 which, in turn, exerts its suppressive effect through either autocrine or paracrine mechanism. To test this hypothesis, we carried out siRNA-mediated gene silencing experiments. Specific siRNA targeting human WISP-1/CCN4 and scrambled control siRNA were introduced into FF2441-NICD1–GFP cells respectively by transient transfection. Knock-down of WISP-1/CCN4 expression was confirmed by immunoblotting analyses 48 hours after transfection (Figure 8A). The siRNAs-transfected FF2441-NICD1–GFP cells (wherin Notch is constitutively activated) were subjected to MTT assay to examine cell growth rate. We observed that specific interference with the WISP-1/CCN4 expression partially, yet significantly relieves the inhibitory effect of Notch activation on cell growth (Figure 8B). The cell growth rate of FF2441-NICD1–GFP cells transfected with specific WISP-1 siRNA, but not control siRNA, was partially restored, suggesting that WISP-1/CCN4 is one of the critical functional mediators of Notch signaling in regulating fibroblast proliferation. These data demonstrated that inhibitory effects of Notch signaling on human fibroblast proliferation are mediated in part by WISP-1/CCN4. That is, WISP-1/CCN4 was demonstrated to be one of functional down-stream targets of Notch signaling in fibroblasts.

### Notch Signaling-Induced WISP-1/CCN4 Expression Is Wnt1-Independent, but Wnt11-Dependent in Fibroblasts

**Figure 9:**
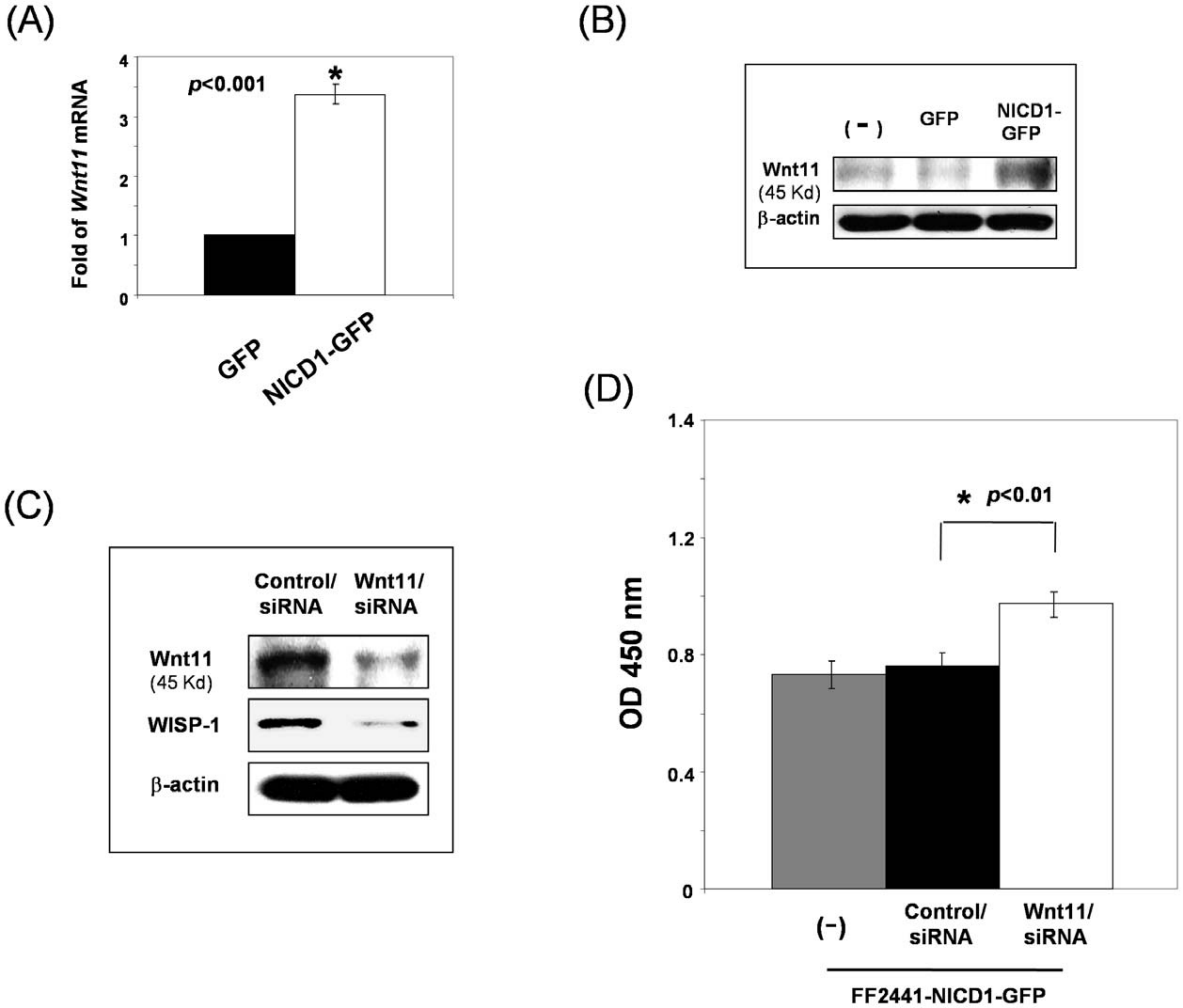
Notch-induced WISP-1/CCN expression is Wnt11-dependent in the fibroblasts. (A) Levels of mRNA of *Wnt11* were up-regulated in FF2441-NICD1-GFP cells compared to FF2441-GFP cells. Data are from RT^2^ PCR Array and presented as fold changed in gene expression by setting levels of genes in FF2441-GFP as “1”. (**B**) Expression of Wnt11 protein was up-regulated by Notch pathway activation in fibroblasts. Expression of Wnt11 in FF2441-NICD1-GFP versus FF2441-GFP and untreated FF2441 (−) cells was analyzed by Western blotting assay. β-actin was used as loading control. (**C**) Blockage of Wnt11 expression resulted in decreased expression of WISP-1/CCN expression. siRNA-mediated knocking-down Wnt11 expression in human fibroblasts. Expression of WISP-1/CCN4 in *wnt11*siRNA-transfected versus non-targeting control siRNA-transfected FF2441-NICD1-GFP cells was analyzed by Western blotting assay. β-actin was used as loading control. (**D**) Blockage of Wnt11 expression relieved Notch's inhibitory effect on cell growth of fibroblasts. Cell proliferation of *wnt11* siRNA-transfected-, non-targeting control siRNA-transfected- and their “parental” FF2441-NICD1-GFP was determined by MTT assays. 2,000 cells/well were plated. Data are presented as mean ± SD of three independently performed experiments.

WISP-1/CCN4 was originally reported to be induced by Wnt1 [18]. However, our PCR array studies did not show that the levels of *Wnt1* gene in FF2441-NICD1–GFP are elevated compared to that in FF2441-GFP cells. Immunoblotting analysis confirmed the PCR array findings on Wnt1 expression (data not shown). Alternatively, we observed that the expression of *Wnt11* gene was up-regulated >3-fold by Notch pathway activation (Figure 9A). Immunoblotting analyses confirmed these findings on the protein levels (Figure 9B). To explore whether Wnt11 is responsible for the induction of WISP-1/CCN4 in human fibroblasts, we blocked Wnt11 expression by siRNA approach and investigated whether inhibition of Wnt-11 expression results in the modulation of WISP-1/CCN4 expression in FF2441-NICD1–GFP cells. As shown in Figure 9C, the upregulation of WISP-1/CCN4 was reversed in FF2441-NICD1–GFP cells when Wnt11 was inhibited by siRNA. As a consequence, cell growth in FF2441-NICD1–GFP cells was rescued (Figure 9D). These data strongly suggest that Notch signaling-induced WISP-1/CCN4 expression in human fibroblasts is mediated through Wnt11, but not Wnt1. Thus, these data identified a novel Wnt1-independent, but Wnt11-dependent mechanism for the induction of WISP-1/CCN expression in fibroblasts and thereby, for the first time, established a linkage between Notch, Wnt11 and WISP-1/CCN4.

## Discussion

Cell cycle and cell growth of fibroblasts are tightly controlled. In normal uninjured tissues, resident fibroblasts are maintained in a relatively quiescent state. When tissue is injured or undergoing tumorigenesis, quiescent resident fibroblasts are stimulated by inflammatory factors or tumor cell-derived stimulatory factors, and activated. The involvement of Notch signaling in regulating cell growth of fibroblasts was previously mostly unexplored. A few prior studies provide inconsistent results with respect to Notch's role in regulating the fibroblast cell cycle. For example, a study showed that Notch1 activation promotes the G_1_-S transition of the cell cycle by inducing the expression of Skp2 and the consequent degradation of the CDK inhibitor p27 in 3T3 mouse fibroblasts [19]. In contrast, activation of Notch signaling via either overexpression of NICD or stabilization of NICD by ablation of SEL-10 (Fbxw7), a negative regulator of Notch signaling, resulted in cell cycle arrest and apoptosis in mouse embryonic fibroblasts (MEFs) [20]. The results of the latter study are consistent with our findings. In other prior work also consistent with our findings, it was reported that inhibition of Notch signaling by soluble forms of the Dll1 and Jagged1 ligands was able to induce fibroblast growth factor receptor (FGFR)-dependent transformation of NIH 3T3 fibroblasts *in vitro* [21]. These studies and ours point to Notch signaling as a negative regulator or ‘break’ on fibroblast cell growth.

An overall assessment of the prior literature and our novel findings appears to indicate that the status of Notch signaling is tightly correlated with the growth characteristics of fibroblasts. Deletion or inhibition of Notch1 signaling exempts fibroblasts from at least one growth control mechanism and cells proliferate faster as occurred in Notch1-deficient (*Notch1* ^−/−^) MEFs, whereas enforced activation of the Notch pathway inhibits cell growth of human dermal fibroblasts. It is, therefore, postulated that deregulation of Notch signaling may be involved in the pathophysiology of some disorders in which fibroblasts are involved. Our study employing Notch-engineered fibroblasts as stromal cells for modulating tumor cell growth/invasion provides an example to demonstrate the biological relevance of Notch activation in fibroblasts. It also suggests a paracrine effect of “Notch-activated” fibroblasts on other types of cells. In this regard, we have recently demonstrated that Notch-induced WISP-1 expression in fibroblasts is responsible for the inhibition of melanoma growth [22]. Our work implies that Notch pathway is likely inactivated in CAFs of tumors, such as melanoma, since “Notch-activated” fibroblasts suppress melanoma cell growth/invasion. This new concept has not been previously tested. Future studies will be required to determine the potential correlation between the status of the Notch activation and biological function of CAFs in tumors.

Our work also revealed a previously unexplored concept. Constitutive activation of Notch1 pathway induces activation of pan-Notch pathways. It indicates existence of a mechanism for Notch signaling undergoing “self propagation”. It is, however, unclear whether this is a unique phenomenon in fibroblasts, or it is a fairly universal mechanism for Notch signaling in other cell types. Although more studies are required, the latter appears to be the case, since we have found that overexpression of NICD1 in other cells, for example, human melanocytes, can induce the expression of several Notch ligands and receptors (ZL unpublished data). Ross et al. also observed that activation of Notch signaling induces Jagged-1 expression in C2C12 and NIH3T3 cells [23]. However, unlike the concept of self-propagation of initial signal, as suggested by our data, it was speculated that the induced Jagged-1 has no apparent authorizing effects on Notch signaling but can promote signaling in naïve cells. That is, it was previously attributed to a mechanism through which Notch signaling can be relayed from cell to cell.

A previously unknown signaling mechanism discovered by this work is the identification of WISP-1/CCN4 as one of functional mediators in delivery of the inhibitory effect of Notch signaling on fibroblast proliferation. The data not only locate WISP-1/CCN4 as a down- stream target gene of Notch signaling, but also unveil a new target for potential therapeutic manipulation. The molecular mechanism regarding how WISP-1/CCN4 exerts its serum-dependent inhibitory effect on cell growth of fibroblasts remains an open question for future study. WISP-1 has been reported to function as a pro-mitogenic factor in mediating TNF-α-induced cardiac fibroblast proliferation [24]. The reason as to why WISP-1 exerts paradoxical biological effects remains unknown. Presumably, it is determined by other cooperative signaling(s) induced by up-stream signal, because TNF-α stimulates cell growth of cardiac fibroblasts whereas Notch signaling suppresses fibroblast proliferation. Alternatively, it is simply cell type-dependent. It has been reported that WISP-1 binding to human skin fibroblasts is mediated through interaction with cell surface decorin and biglycan [25]. It may prove worthwhile to investigate whether decorin and biglycan are responsible for mediating WISP-1's action on fibroblast growth inhibition.

WISP-1 was initially identified as a Wnt1 responsive target [18], and belongs to the CCN family, which includes connective tissue growth factor (CTGF), cysteine-rich-61 (CYR61), and nephroblastoma overexpressed (NOV) [26]. NOV (CCN3) has been shown to associate with Notch1 extracellular domain and exert a positive effect on Notch signaling in inhibiting myoblasts differentiation [27], while our findings reveal that WISP-1/CCN4 functions as a down-stream target of Notch signaling in fibroblasts. These studies provide representative examples to demonstrate the interaction between Notch and CCN family. Until now, Wnt1 is the only member in the Wnt family known to induce WISP-1. Wnt4 is unable to up-regulate WISP-1 expression in the same experimental setting as Wnt1 does [18]. The promoter of WISP-1 has been shown to be activated by both Wnt 1 and β-catenin expression. TCF/LEF sites played a minor role, whereas the CREB site played an important role in the transcriptional activation [28]. Our observation that WISP-1 responds to Wnt11, but not Wnt1, mediates the observed effects, expands the scope of the Wnt family members involved in regulating WISP-1 and suggests that the Wnt/WISP axis may be cell type-specific. However, unlike Wnt1, which is known to activate the canonical Wnt/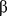-catenin pathway, Wnt11 is classified as a non-canonical signaling. Wnt11 is essential for the development of the heart and kidney [29]–[32], and is also implicated in cancer [33]–[36]. Wnt11 signaling is thought to function in part by inhibiting the activity of the β-catenin-dependent Wnt pathway [37]. We have not evaluated the activity of β-catenin in response to Wnt11 signaling in this study. Future work will be required to elucidate the Wnt11 down-stream pathway responsible for regulating WISP-1 expression in fibroblasts.

In summary, we herein report an inhibitory role of Notch signaling in regulating cell growth of fibroblasts, which is partially mediated by the induction of WISP-1/CCN4 through a Wnt11-dependent, but Wnt1-independent mechanism. Our study establishes a functional linkage between Notch, Wnt11 and WISP-1/CCN4, and suggests a central role for Notch in coordination between these. The new findings lead us to postulate that Notch signaling in fibroblasts could potentially be implicated in some pathologic states featuring fibroblast growth deregulation.

## Methods

### Reagents

Recombinant human WISP-1/CCN4 was purchased from R & D Systems (Minneapolis, MN). SDS-polyacrylamide gels were obtained from Invitrogen (Carlsbad, CA). X-ray films were purchased from Kodak (Rochester, NY). All other chemicals and solutions were from Sigma–Aldrich (St. Louis, MO) unless otherwise indicated.

### Mice and Cells

*Notch1^Flox/Flox^* mice were established as described previously [38]. Animal experiments were approved by the Institute Animal Care and Use Committee of the University of Miami (IACUC #10-228). Primary human dermal fibroblasts (FF2441) were initiated as explant cultures from trypsin-treated and epidermis-stripped neonatal foreskin [14] and cultured in Dulbecco's Modified Eagle's Medium (DMEM) supplemented with 20 mM L-glutamine (Invitrogen), 8 mM HEPES and 10% FBS (Hyclone, Logan, UT). The MEFs were isolated from *Notch1^Flox/Flox^* mice using the method described previously [39] and cultured in DMEM with glutamine, HEPES and 10% FBS. All experiments were performed with MEFs at passage 3–10. Human metastatic melanoma cell line, 1205Lu (ATCC, #CRL-2812), was cultured in 2% FBS-W489 medium as described [14]. All cells were incubated at 37°C in 98% humidified air containing 5% CO_2_.

### Recombinant Lentiviruses and Retroviruses

Methods for generation of GFP/lenti and NICD1–GFP/lenti were described previously [15]. Cre/lenti was constructed by inserting *Cre* gene into pHX' lentiviral vector [15]. Production of pseudotyped lentivirus was achieved by co-transfecting 293 T cells (ATCC # CRL-11268™) with three plasmids as described [15]. The lentiviruses collected 48 hours post-transfection displayed titers of around 10^7^ transducing units/ml in NIH/3T3 cells (ATCC, #CRL-1658™). Retroviral vector MAML305/pBabe (DN-MAML1 (Myc-tagged)) and empty pBabe vector (Mock) were described previously [40]. To infect target cells by lentiviruses and retroviruses, cells were exposed six hours to virus with MOI (multiplicity of infection) 5 in the presence of 4 µg/ml polybrene. Cells were then washed, cultured with regular complete medium for two additional days, and analyzed for protein expression by Western blot or pooled for subsequent analysis as indicated in individual experiments.

### PCR Array

The Human Notch Signaling Pathway RT° Profiler™ PCR Array (# PAHS-059, SABiosciences, Frederick, MD) and Human Cell Cycle RT° Profiler™ PCR Array (#PAHS-020) quantitatively profiles the expression of 84 genes involved in Notch signaling and 84 genes involved in cell cycle control, respectively. Total RNA was extracted from cells using Trizol® (Invitrogen) and cDNA was synthesized using RT° First Strand Kits (SABiosciences). PCR array was carried out according to the manufacturer's protocol. The threshold cycle (Ct) values were used to plot a standard curve. All samples were normalized to the relative levels of *β-actin*, and results are expressed as fluorescence intensity in relative levels.

### Immunoblotting

Western blotting was performed as described [15]. Membranes were probed with Abs to Notch1 (a rabbit polyclonal antiserum directly against residues 1759–2095) [15], cleaved/activated Notch1 (an antibody recognizing Val1744 (ab52301, Abcam, Cambridge, MA)), phospho-MAPK (#9106, Cell Signaling Technologies, Danvers, MA), p44/42 MAPK (#9102, Cell Signaling Technologies), WISP-1/CCN3 (H-57, sc-25441, Santa Cruz Biotechnologies, Santa Cruz, CA), Wnt-11 (sc-50360, Santa Cruz Biotechnologies), Hey-1 (GTX42614, GeneTex, Irvine, CA) or β-actin (AC-15, Abcam). Myc-tagged DN-MAML1 was detected on Western blot by 9-B11 Ab (Santa Cruz). This was followed by probing with HRP-conjugated second Ab (Jackson Immunoresearch, PA) and subjected to ECL (Amersham Biosciences, Piscataway, NJ). Membranes were stripped and re-blotted as required in the individual experiment. To quantify the bands in the blots, autophotographs of Western blots were scanned by computerized densitometry (Molecular Dynamics).

### Construction of *WISP1 Promoter*-*Luc2*/Lentiviral Vector and Luciferase Assay

Plasmids containing *WISP1 promoter*-*Luc2* fragment (WISP1 PROM_01, ID: S113793) and two control fragments (RPL10PROM_01, ID: S108908, and R01_PROM, ID: S190001) were purchased from SwitchGear Genomics (Menlo Park, CA). WISP1 PROM_01 was digested by SacI and SalI, and both RPL10PROM_01 and R01_PROM were digested by MulI and SalI, respectively. ∼2.1 kb WISP1 promoter-Luc2 and two control fragments were isolated, blunted and inserted into SmaI cut pHX' vector. Cells were transduced with *WISP1 promoter*-*Luc2*/Lentivirus, control RPL10PROM/Lentivirus and R01_PROM/Lentivirus, respectively. 48 hours post-transduction, 2×10^4^ cells/well in 96 well plates (triplicates) were measured for luciferase activity. Luciferase assays were performed using Steady-Glo® Luciferase Assay kit (Promega, Madison, WI) according to the manufacturer's protocol. Luciferase activity measurement was corrected by subtraction of readings of controls.

### MTT Assay

Cell growth was measured by MTT assay. MTT cell proliferation kits were purchased from BioVision Technologies (Exton, PA). Cell proliferation was measured according to the manufacturer's protocol. 2,000–5,000 cells/well were cultured in 96-well plate and cultured in DMEM with or without FBS as indicated in individual experiment. Samples were assayed in triplicate and experiments were repeated three times.

### Apoptosis Assay

Cell apoptosis was detected by TUNEL based ApopTag® kit (S7100) from Chemicon (Billerica, MA) according to the manufacturer's protocol. Briefly, cells fixed with 4% formaldehyde were pre-treated with 3% H_2_O_2_ for 5 minutes at room temperature followed by washing with PBS. After incubation with equilibration buffer for 15 minutes at room temperature and TdT enzyme at 37°C for 60 minutes, cells were treated with stop/wash buffer for 10 minutes after being agitated for 15 seconds at room temperature, followed by washing with PBS. Cells were then incubated with anti-digoxigenin conjugate for 30 minutes at room temperature followed by washing with PBS. Apoptotic cells were stained with colorimetric substrates diaminobenzidine (DAB). Serum-starved cells (72 hours) were used as positive control.

### In vitro Cell Motility Assay and Time-Lapse Photography

To measure cell motility *in vitro*, MEF/Cre and MEF/GFP cells were cultured overnight to sub-confluence in 24-well plate. A gap was created by scratching cells with a standard tip. Velocity of fibroblast migration (µM per minute) was recorded using time-lapse photography at 10-minute intervals, for 20 hours. Fifteen randomly selected individual cells per well were tracked, and data were analyzed by ImagePro 5.0 software (MediaCybernetics, Silver Spring, MD).

### In vitro Three-Dimensional (3D) Skin Melanoma Model

3D skin melanoma model was prepared as described [16], [17]. A total of 3 mL of fibroblasts (FF2441-NICD1–GFP versus FF2441-GFP, 7.5×10^4^cells/mL) in a 4∶1 mixture of bovine type I collagen (Organogenesis, Canton, MA): Matrigel® (BD Bioscience) was added to each insert of tissue culture trays (Organogenesis) and were allowed to constrict in DMEM with 10% FBS for 7 days at 37°C. For epidermal reconstruction, human keratinocytes, isolated from human epidermis of neonatal foreskins and cultured as described, [17], [41] were mixed with human metastatic melanoma cells (1205Lu) at a ratio of 5∶1 in epidermal growth medium composed of three parts DMEM and 1 part Ham's F-12 supplemented with 2.4 M CaCl_2_, 0.18 mM adenine, 4 mM glutamine, 10 mg/ml selenium, 10 µM ethanolamine, 0.1 mM O-phosphoryl ethanolamine, 10 µg/mL insulin, 10 µg/mL transferrin, 20 pM tri-iodothyronine, 0.5 µg/mL hydrocortisone and 4 pM progesterone. A total of 5×10^6^ cells were seeded on each contracted collagen gel. Cultures were kept submerged in medium containing 1 ng/mL EGF and 0.1% dialysed newborn calf serum for 2 days, then in 0.2 ng/mL EGF and 0.1% dialysed newborn calf serum for another 2 days, and then were raised to the air–liquid interface via feeding from below with medium containing 2% dialysed newborn calf serum. After 14 days, skin reconstructs were fixed with 4% paraformaldehyde and were embedded in paraffin. Cell growth of melanoma cells was measured by counting melanoma cell number per low power field (LPF, X10). The invasive capacity of melanoma cells was determined by measuring the % of area of melanoma cells occupied in a given LPF based on morphological evaluation using H&E staining. Data are calculated based on that from 5 randomly selected fields/section and totally 10 sections per group.

### siRNA Gene Silencing

Short interfering RNA (siRNA) targeting the human form of WISP-1/CCN4 (sc-39335), Wnt-11 (sc-41120) and control, nontargeting siRNA (sc-37007), along with Transfection Reagent (sc-29528), siRNA Transfection Medium (sc-36868) and siRNA Dilution Buffer (sc-29527) were purchased from Santa Cruz Biotechnology. The experiments were performed according to the manufacturer's protocol.

### Statistical Analysis

All data is expressed as mean ± SD. Statistical analysis was carried out using paired Student's *t*-test. Values considered statistically significant were *P*<0.05.
